# Stepped care treatment for depression and anxiety in primary care. a randomized controlled trial

**DOI:** 10.1186/1745-6215-12-171

**Published:** 2011-07-07

**Authors:** Wike Seekles, Annemieke van Straten, Aartjan Beekman, Harm van Marwijk, Pim Cuijpers

**Affiliations:** 1 Department of Clinical Psychology, VU University, Amsterdam, The Netherlands; 2 Department of Psychiatry, VU University Medical Centre, Amsterdam, The Netherlands; 3 Department of General Practice, VU University Medical Centre, Amsterdam, The Netherlands; 4 EMGO institute for Health Care and Research, VU University Medical Centre, Amsterdam, The Netherlands

## Abstract

**Background:**

Depressive and anxiety disorders are common in general practice but not always treated adequately. Introducing stepped care might improve this. In this randomized trial we examined the effectiveness of such a stepped care model.

**Methods:**

The study population consisted of primary care attendees aged 18-65 years with minor or major DSM-IV depressive and/or anxiety disorders, recruited through screening. We randomized 120 patients to either stepped care or care as usual. The stepped care program consisted of (1) *watchful waiting*, (2) *guided self-help*, (3) short face-to-face *Problem Solving Treatment *and (4) *pharmacotherapy and/or specialized mental health care*. Patients were assessed at baseline and after 8, 16 and 24 weeks.

**Results:**

Symptoms of depression and anxiety decreased significantly over time for both groups. However, there was no statistically significant difference between the two groups (IDS: *P *= 0.35 and HADS: *P *= 0.64). The largest, but not significant, effect (*d *= -0.21) was found for anxiety on T3. In both groups approximately 48% of the patients were recovered from their DSM-IV diagnosis at the final 6 months assessment.

**Conclusions:**

In summary we could not demonstrate that stepped care for depression and anxiety in general practice was more effective than care as usual. Possible reasons are discussed.

**Trial Registration:**

Current Controlled Trails: ISRCTN17831610.

## Background

Depressive and anxiety disorders are common in general practice [1]. These disorders are often associated with serious functional impairment, reduced quality of life [2,3], high levels of service use and rising economic costs [4,5]. Persons with depressive and anxiety disorders often seek help in primary care [6]. Although evidence based clinical guidelines are available for the treatment of depressive and anxiety disorders in primary care [7,8], initiation of, and adherence to effective treatment is usually poor [9-12]. An important problem is the underrecognition of these disorders [13]. For depression approximately half of all patients are not recognized by their general practitioner (GP) as having psychological problems [14,15]. For anxiety disorders this is about 75% [16]. Another problem is that most patients who do receive treatment, receive antidepressants [17-19], whilst the majority of primary care patients prefer psychotherapy as a treatment [20]. Furthermore, antidepressants are often prescribed in relatively mild cases even though it has been established that medication is not effective in these patients [21,22]. Given these problems and the fact that depressive and anxiety disorders have a high burden of disease, there is a need for better managed and structured treatment in primary care.

Recent studies of treating depression and anxiety have proposed several models of disease management [23], collaborative care [24] and stepped care [25]. One core element of these models is the presence of a care manager who is responsible for managing the care that the patient receives. Another core element is the establishment of a more integrated cooperation between primary care and specialized mental health care [26]. The stepped care model could provide a solution for the problem of applying effective, evidence-based care for depression and anxiety in primary care through its objective of initiating interventions at the right time and as adequately as possible. Care is offered not earlier or more intensely than necessary and not later or less intensely than needed [25,27]. In a stepped care model, all eligible patients start with an evidence-based treatment of low intensity as a first step. Progress is monitored and patients who do not respond adequately can 'step up' to a subsequent treatment of higher intensity [28]. This model is suitable in (primary) mental health care because the proposed treatments in the most recent depression guidelines, published in the Netherlands in 2009, range from less intensive interventions like psychoeducation or self help interventions (individual or group courses), problem solving treatment (PST), to more intensive treatments such as cognitive behavioral therapy and pharmacotherapy.

Another important feature of the stepped care model is that the model is self-correcting. Self-correcting means that the results of treatments and decisions about treatment provisions are monitored systematically and necessary changes are made ('stepping up') if current treatments are not achieving significant health improvement [25]. A care manager coordinates a stepped care program, preferably a nurse or social worker who supports the primary care clinic handling psychiatric problems. In The Netherlands the most likely candidate for this role is a psychiatric nurse working in primary care. This care manager monitors the patients, provides the first treatments in the stepped care model and refers the patient to the appropriate mental health care specialist if necessary.

Stepped care models have been developed for different health problems, for example eating disorders [29,30], alcohol related disorders [31,32], smoking cessation [33] and prevention of anxiety and mood disorders in elderly [34]. At present stepped care is recommended for health care in several guidelines, for example in the NICE guidelines for anxiety, depression and obsessive compulsive disorder and in the depression guidelines in the UK (NHS) [35-37] and by the ministry of health in New Zealand in 2009 [38]. Although there is some supportive evidence for stepped care, there are few randomised trials to demonstrate convincing evidence and evaluations of this program.

The aim of this study is to examine the effectiveness of a stepped care model in primary care via a randomized controlled trial for patients with depressive and/or anxiety disorders. We will examine the reduction of symptoms, recovery and the speed of recovery.

## Methods

### Study design

The methods of this study have been published previously [39]. In short, 120 participants were recruited through 32 primary care practices. They were randomly assigned into two groups: stepped care or care as usual. We chose a pragmatic design because we wanted to study the effects of the intervention among typical patients in a real-life setting because this increases external validity [40,41]. This means that we implemented a stepped care model in general practice in collaboration with specialized mental health care centres. Treatment of mental health in primary care is common in The Netherlands. A recent development is to establish a psychiatric nurse or psychologist in general practice. The GP can refer patients within his own practice to a professional instead of direct referral to specialized mental health care. Inclusion took place between April 2007 and May 2008. The study was approved by the Medical Ethical Committee of the VU Medical Center and all participants gave written, informed consent. The effectiveness of the first step of this stepped care model (guided self-help) has been reported separately [42]. Self-help and bibliotherapy seem promising low intensity treatments for depression and anxiety in several studies, but there are few studies that report results on guided-self help in general practice, therefore these relevant results are reported separately, with the focus on self-help/bibliotherapy in primary care. These data were derived from a larger project (stepped care) and the outcomes of this whole project are reported in this paper.

### Inclusion and exclusion criteria

We included adults aged 18-65 years with one or more of the following DSM-IV [43] diagnoses: major depression (single episode or recurrent), dysthymia, panic disorder (with or without agoraphobia), social phobia or generalized anxiety disorder. We also included patients with a minor depression or a minor anxiety disorder. DSM-IV research criteria were used to define minor depression. For minor depression only two to four out of the nine DSM-IV symptoms had to be present, of which at least one had to be a core symptom (depressed mood or markedly diminished interest or pleasure in all, or almost all, activities). As there are no DSM-IV criteria for minor anxiety we defined this as a score of 12 or more on the Hospital Anxiety and Depression Scale (HADS) [44] and dysfunctioning in daily life (household, work, social relations and/or social activities). Patients were excluded in case of a psychotic or bipolar disorder, current (< 2 months) treatment (medical/psychotherapy) for psychological problems, prominent suicide ideation, severe alcohol problems (> 20 on the Alcohol Use Disorders Identification Test (AUDIT) [45], no motivation for treatment or insufficient knowledge of the Dutch language.

### Recruitment

#### Recruitment of GPs

In this study we collaborated with two mental health centres in Amsterdam (GGZ inGeest and Mentrum). Both of these mental health centres employ psychiatric nurses and psychologists, who work for a few hours per week in a GP practice. Usually, GPs refer patients to these psychiatric nurses/psychologists for short-term treatments. First we approached the psychiatric nurses and psychologists and after they consented we invited the corresponding GPs to collaborate in this study. In total we included 32 GPs in 18 general practices.

#### Recruitment of patients

Patients were recruited by sending all patients of the participating GPs a screening questionnaire. All patients with a positive screener for depression and/or anxiety were assigned to a watchful waiting period of 4 weeks. After 4 weeks all patients were screened again to exclude the patients who recovered spontaneously. This second screener was included in the baseline questionnaire (T0) and was sent to all patients together with general information about the project and an informed consent form. All eligible patients were approached for a diagnostic interview (Composite International Diagnostic Interview (CIDI) [46] by telephone to check the in- and exclusion criteria. Patients who met the inclusion criteria *and *returned their informed consent were randomized. One hundred twenty patients were included.

#### Number of patients

**Figure 1:**
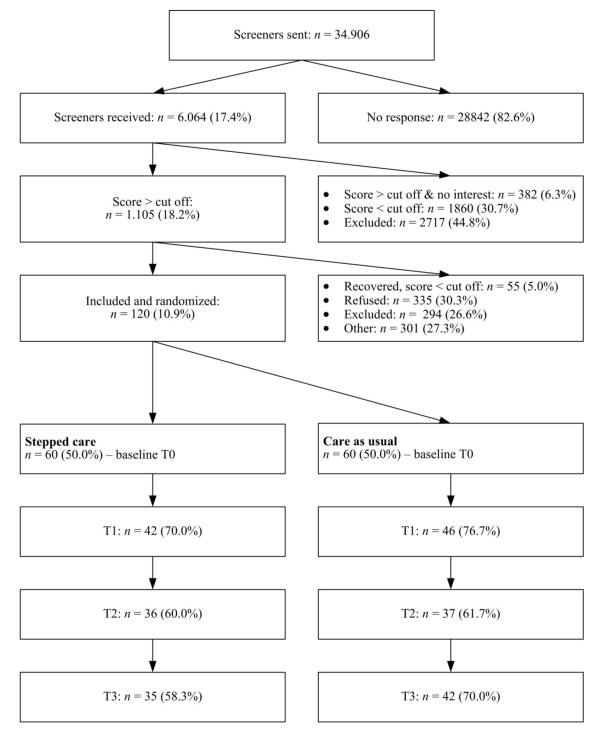
**Flowchart inclusion and randomization**.

Before the start of the study we calculated that we needed 2*100 patients in order to be able to detect clinically relevant difference (*d *= 0.40) with a power of 80% and an alpha of 0.05 [47]. However, inclusion was slower than we expected and therefore we included only 120 patients [Figure 1]. In total we send 34.906 screeners and had a response of 17.4%. Of the 1105 patients who scored positively on the first screener, 335 (30.3%) declined to participate any further and 301 (27.3%) could not be reached or did not respond. Of the remaining 469 patients, 294 (62.6%) were excluded (most given exclusion reason: current or recent (< 2 months) psychological or pharmacological treatment), 55 patients (11.7%) were recovered and scored negatively on the second screener and 120 patients (25.6%) were included.

### Randomization

We randomized patients at an individual level. They were randomized into two groups and we used blocks of 4 to prevent overburdening the care managers. Care managers were not informed about patients who were randomized to care as usual. An independent researcher, not involved in the current project, used computer generated block randomization to produce sealed envelopes. After every inclusion the researcher opened a sealed envelope. We randomized 60 patients to the stepped care program and 60 patients to care as usual.

### Intervention

The stepped care intervention consisted of four steps: *(1) watchful waiting*. The patients received no treatment for four weeks. In this project, as mentioned earlier, only patients who still showed symptoms of depression and/or anxiety after the watchful waiting period were included. The included patients started with (2) *guided self-help*. Self-help can be defined as a standardized psychological treatment that a patient can work through on his/her own, possibly with some guidance [48]. Most self-help interventions are based on cognitive-behavioural therapy (CBT) [49] but nowadays other types of treatment (i.e. problem solving treatment (PST), interpersonal psychotherapy (IPT) have become available as (guided) self-help interventions as well. Self-help interventions are available via books (bibliotherapy) and via the computer (web-based, CD-ROM, DVD) and they can be pure self-help or guided self-help. In this first step guided self-help was introduced in a 30 minute session with a care manager. This session enabled the care manager to check exclusion criteria, give psychoeducation (*e.g*., advice on lifestyle) and to explain the self-help interventions. In this study we used two different self-help interventions. The first was a generic intervention based on problem solving treatment. Previous studies have demonstrated the effectiveness of this intervention among people in general population with symptoms of depression and/or anxiety [50,51]. This intervention was available as a book and through the Internet. The patient could choose to get feedback by email or by telephone. The feedback was given by junior psychologists. They were trained by the senior researcher (AvS). The feedback is not therapeutic in nature and was primarily aimed at helping people to understand the techniques which are offered in the course. Furthermore, the feedback is used to motivate people to continue the course. The feedback is designed as being easy to learn by, for example, a care manager or psychiatric nurse. The second self-help intervention was specifically aimed at patients with phobias and was based on exposure therapy. This course also took six weeks to complete and was only available in book form. Feedback was therefore provided by telephone. During the first face-to-face session with the care manager it was decided which self-help course was most suitable. Patients who did not recover from self-help treatment started with (3) face-to-face *Problem Solving Treatment (PST)*. PST is a short psychological intervention, 5 sessions of 45 minutes each, provided by the care manager in the primary care practice [52]. The treatment protocol was based on the protocol as described by Mynors-Wallis [53]. Patients who were unresponsive to this treatment proceeded to the last step of the stepped care program (4) *pharmacotherapy and/or referral for specialized mental health care*. When patients did not recover from PST they had one more session with the care manager and discussed the next step: either pharmacotherapy or more specialized mental health care. The care manager was responsible for setting up the next meeting (either with the GP or in mental health care). Before the start of the study it was agreed with the specialized mental health care centers that patients from this study could directly start with psychological treatment. They could skip normal intake procedures as well as waiting lists.

#### Exceptions

Even though there is no clear evidence that patients with more severe symptoms of anxiety or depression do not benefit from low intensity (self-help) interventions, we decided that patients with more severe disorders should be referred to more specialized mental health care and/or pharmacotherapy directly and skip the preceding steps. Severity of the disorders was based on questions about daily functioning on the Work and Social Adjustment Scale (WSAS) [54]. If the patient experienced extreme dysfunctioning (score of 8 or higher) on minimal three of the four domains (household, work, social relations and social activities) he/she was directed immediately to the fourth step of the stepped care program.

#### Care as Usual

Patients randomized to the 'care as usual' were advised to see their GP to discuss treatment options. The GP is very easy accessible in The Netherlands, due to good health insurance for everyone. Most patients go to their GP with mental health complaints. The GP is a gatekeeper for secondary (specialized) mental health care.

#### Assessments and definition of recovery

After each step in the stepped care intervention, *i.e*. after every 8 weeks, patients were monitored. We assessed symptoms of depression and anxiety as well as daily functioning. We considered a patient to be recovered when he/she scored less than 14 on the Inventory of Depressive Symptomatology (IDS) [55], *and *scored less than 8 on the HADS [44]*and *scored less than 6 on the WSAS [54]. This criterion was based on several studies [55-58].

### Instruments

#### Depressive symptoms

We used the Inventory of Depressive Symptomatology (IDS) to measure depressive symptoms. The IDS consists of 30 items and the total score varies between 0 and 79. Scores below 14 indicate an absence of depressive symptoms. We used this cut-off score as an indication for recovery from depressive symptoms [55,56]. Internal consistency is high for the IDS (Cronbach's alpha: 0.92) [55].

#### Anxiety symptoms

For identifying anxiety symptoms we used the Hospital Anxiety and Depression Scale (HADS) [44] which is designed to identify anxiety disorders among patients in non psychiatric settings. The HADS consists of 7 items. Item responses are on a 0 to 4 scale (0 = "none" and higher ratings reflect greater degrees of symptom severity). Total scores range from 0 to 21. The HADS showed good homogeneity and reliability, with Cronbach's alpha ranging from 0.81 to 0.84 in various clinical and non-clinical Dutch samples [59].

#### Dysfunction

We measured daily functioning of the patient via four questions on the Work and Social Adjustment Scale (WSAS) [60]. Using this questionnaire, the patient gives an estimate, on a scale from 1 to 10, of the perceived dysfunctioning in his or her daily life. The questions contain four domains: household tasks, work, social relations and social activities.

#### Composite International Diagnostic Interview (CIDI)

The CIDI (version 2.1), a structured interview developed by the World Health Organisation [46], enables trained interviewers to assess psychiatric diagnosis defined in the DSM-IV [43]. The assessment typically lasts 30 to 75 minutes, depending on the mental state of the respondents [61]. In this study, current mental status (last six months) is taken into consideration. The interviews were conducted by psychology master students who had followed a CIDI-training. Interviewers were blind regarding randomization at the follow-up assessment.

### Statistical Analysis

All analyses were conducted according to the intention-to-treat principle. Missing data were imputed using regression imputation, except for the Generalized Estimating Equations (GEE)-analysis because imputation is part of the analysis. The effectiveness of stepped care compared to care as usual is expressed in different ways. First we used t-tests to determine if there were any statistically significant differences between the test scores on every assessment of the two groups, to see if they showed any statistically differences (*P*-values). Second, we calculated effect sizes (Cohen's *d *[47]). This was done for every assessment by subtracting the post-test mean score of the control group from the post-test mean of the intervention group. This difference in mean scores is divided by the pooled, over time and group, standard deviation. A Cohen's *d *of 0.5 thus indicates that the mean of the intervention group is half a standard deviation larger than the mean of the control group. Values of *d *from 0.56 to 1.2 can be assumed to be large, 0.33 to 0.55 are moderate, and 0 to 0.32 are small [62]. Second, we assessed the differences between the assessments by means of Generalized Estimating Equations (GEE) [63]. The method of GEE is often used to analyze longitudinal and other correlated response data. GEE takes into account the correlational nature of repeated measures data within subjects, and securing minimal loss of patients due to incomplete data. We included time as a continuous variable. And third, we examined differences in the percentages of patients who recovered from their diagnosis at the last assessment.

## Results

### Baseline characteristics

Almost two thirds of the included participants were women (65%) and the mean age (SD) was 50.2 (SD = 11.2) years. At baseline, the mean IDS score was 30.6 (SD = 10.8) and the mean HADS score was 9.7 (SD = 4.0) [Table 1]. This is for both depression and anxiety a mild to moderate mean score. Twelve (9.9%) of the 120 randomized participants reported severe dysfunctioning on the WSAS. Of those 12 patients, 5 were randomized to stepped care. Those 5 patients proceeded directly to the fourth step and did not receive a self-help intervention. We also referred 4 patients without severe dysfunctioning to specialized mental health care based on the judgment of the psychiatric nurse. There were no significant differences between the stepped care and control group on any of the demographic or clinical variables.

**Table 1:** Baseline characteristics

	Total *n *(%)	Stepped care *n *(%)	Care as usual *n *(%)	*P*
**Demographics**				
Participants	120 (100%)	60 (100%)	60 (100%)	
Mean age, years (SD)	50.2 (11.2%)	51.2 (9.8%)	49.2 (12.4%)	0.13
Gender (female)	78 (65.0%)	41 (68.3%)	37 (61.7%)	0.44
With a paid job	69 (57.5%)	33 (55.0%)	36 (60.0%)	0.58
Born in The Netherlands	95 (80.5%)	49 (81.7%)	46 (79.3%)	0.50
Married	40 (33.3%)	18 (30.0%)	22 (36.7%)	0.44
**Clinical status**				
Depression (IDS, mean (SD))	30.7 (10.8%)	29.5 (11.3%)	31.8 (10.3%)	0.24
Anxiety (HADS, mean (SD))^a^	9.7 (4.0%)	9.7 (4.1%)	9.8 (4.0%)	0.91
				
**DSM-IV diagnosis**				
*Anxiety*				
Any anxiety disorder	110 (91.7%)	55 (91.7%)	55 (91.7%)	1.00
Only anxiety disorder	51 (42.5%)	30 (50.0%)	21 (35.0%)	0.10
*Depression*				
Any depressive disorder	69 (57.5%)	30 (50.0%)	39 (65.0%)	0.10
Only depressive disorder	10 (8.3%)	5 (8.3%)	5 (8.3%)	1.00
				
Comorbid depressive and anxiety disorder	59 (49.2%)	25 (41.7%)	34 (56.7%)	0.10
Mean age of onset DSM-IV diagnosis (SD)^b^	28.3 (16.0)	28.8 (15.5)	27.8 (16.5)	0.75

HADS = Hospital Anxiety and Depression Scale, IDS = Inventory of Depressive Symptomatology, ^a^*n *= 107 (one missing HADS), ^b^*n *= 100 (eight patients with unofficial DSM-diagnosis)

### Treatment adherence

**Figure 2:**
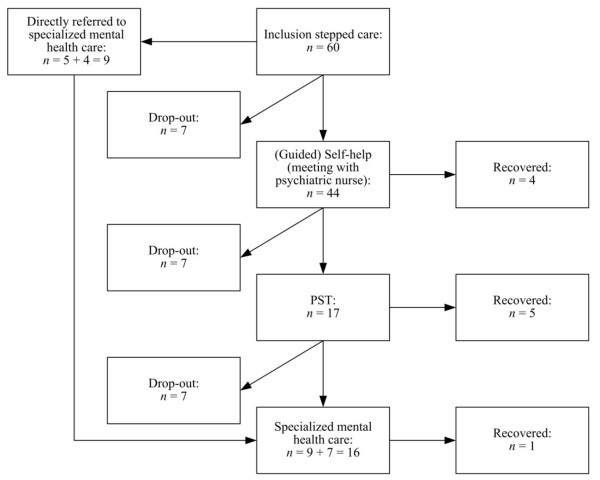
**Stepped care program flow chart**.

Of the 60 stepped care patients, 44 (73.3%) received a first face-to-face meeting with the psychiatric nurse and received a self-help course and 9 (15%) were referred directly after inclusion to the fourth step. There were 7 (11.7%) patients who left the stepped care program (3 were physical ill and 4 could not be reached when the psychiatric nurse tried to make an appointment). After self-help 4 of the 44 (9.1%) patients were recovered and 23 (55.3%) patients left during the program. Different reasons were given for leaving the program, for example they felt better, were physical impaired, they moved, received other care, were disappointed in care or preferred face-to-face treatment. Seventeen of the 44 (40.5%) patients moved to the next step and received PST. Five of 17 (29.4%) left the program during PST (mostly the same reasons were given as after the first step) and did also not want to receive specialized mental health care. After PST 5 (29.4%) patients were recovered. Seven of 17 (41.2%) patients were referred to specialized mental health care after they had run through the whole stepped care program and after referral. For these patients we arranged an intake in specialized mental health care. Four of the seven patients received care in a matching program (for example mood or anxiety). One patient did not want to be referred and two patients did not need specialized mental health care according to themselves in agreement with their psychiatric nurse. One of the 7 referred patients (14.3%) has recovered at time of the last assessment [Figure 2].

GPs were asked to refrain from offering any treatment to patients who were included in the stepped care group (treatment group). Benzodiazepines were allowed in both study groups. Patients in the stepped care group were only allowed to receive antidepressants in later phase of the treatment protocol. Table 2 describes the received care as usual for the patients in this group between the steps of the model. It shows that half of the patients went to see their GP and on average 25% received mental health care. Only one patient (2.4%) received antidepressants during the last step.

**Table 2:** Received treatment in care as usual per assessment

Type of treatment	T0 - T1	T1 - T2	T2 - T3
	*n *= 38	*n *= 37	*n = *42
GP	21 (55.0%)	19 (31.7%)	24 (57.1%)
Mental health care	10 (26.0%)	9 (24.3%)	12 (28.6%)
Medical officer	2 (5.0%)	1 (2.7%)	2 (4.8%)
Social worker	1 (3.0%)	2 (5.4%)	4 (9.5%)
Alternative medicine	4 (11.0%)	2 (5.4%)	1 (2.4%)
Benzodiazepines	9 (24.2%)	7 (18.9%)	3 (7.1%)
Antidepressants	0 (0.0%)	0 (0.0%)	1 (2.4%)

### Treatment effects: effect size

When we compared stepped care and care as usual on IDS on each measurement, we could not demonstrate any significant differences between both groups (T0: *P *= 0.24, T1: *P *= 0.49, T2: *P *= 0.86 and T3: *P *= 0.55). When we compared stepped care and care as usual on the HADS on each measurement, we did not find any significant differences between the groups either (T0: *P *= 0.91, T1: *P *= 0.47, T2: *P *= 0.71 and T3: *P *= 0.22). The largest, but not significant, effect (*d *= 0.21) was found for anxiety on T3 [Table 3].

**Table 3:** Observed means, SDs, p-values and Cohen's d on the IDS and HADS

	Stepped care	Care as usual	*P*	*d*
	*n *= 60	*n *= 60		
**T0**				
IDS	29.5 (11.3)	38.8 (10.3)	0.24	
HADS	9.7 (4.1)	9.8 (4.0)	0.91	
**T1**				
IDS	25.6 (12.3)	27.2 (12.9)	0.49	0.12
HADS	8.7 (4.3)	9.3 (3.8)	0.47	0.14
**T2**				
IDS	25.0 (13.0)	25.4 (11.0)	0.86	0.03
HADS	9.8 (3.8)	9.1 (3.7)	0.71	0.05
**T3**				
IDS	25.0 (12.5)	25.4 (13.0)	0.55	0.11
HADS	7.9 (3.7)	8.8 (4.2)	0.22	0.21

IDS = Inventory of Depressive Symptomatology

HADS = Hospital Anxiety and Depression Scale

### Treatment effect: recovery per assessment

#### Depression (IDS)

GEE-analysis indicated that depressive symptoms significantly decrease over time in both the stepped care and care as usual condition (*P *< .01). But there was no significant difference between both groups (*P *= 0.35) and no interaction effect between time and group (*P *= 0.82) (*B *= 0.17; 95% CI = -1.33 to 1.68).

#### Anxiety (HADS)

GEE-analysis indicated a significant decrease of anxiety symptoms in both stepped care and care as usual over time (*P *< 0.01). But there was no significant difference between both groups (*P *= 0.64) and no interaction effect between time and randomization (p = 0.10) (*B *= -0.45; 95% CI = -0.98 to 0.09).

### Treatment effect: diagnoses

There was no statistically significant difference between the percentage recovered in the stepped care group (*n *= 18; 47.4%) and the care as usual group (*n *= 20; 51.3%; *P *= 0.73). We performed a sensitivity analyses. First we assumed that all patients whose diagnosis was missing had actually recovered (best case scenario). In this case 68.3% of the stepped care patients and 66.7% of the care as usual patients were recovered. There was no difference in the percentage of recovery between the two groups (*P *= 0.85). Second we assumed that all patients whose diagnosis was missing still suffered from a depression or anxiety disorder (worst case scenario). In this case 33.3% of the stepped care patients and 30% of the care as usual patients were recovered. Again, there was no difference in the percentage of recovery between the two groups (*P *= 0.70).

## Discussion

We do not find any evidence that a stepped care model outperformed care as usual. In both groups the levels of symptoms declined, but there were no differences between stepped care and care as usual. In both groups approximately 50% still received a DSM-IV diagnosis six months after inclusion.

There might be several reasons why stepped care did not outperform care as usual in this trial. The following reasons are discussed: recruitment and need for treatment, motivation, mild symptoms, chronicity, adherence and well developed care as usual. First of all, there was little interest in participating in a stepped care model given the difficulty of recruiting patients for this study. The patients that were recruited might have been a select group due to a selection bias through screening. A recent meta-analysis of psychological treatment of depression in primary care [64] demonstrated that studies with recruitment through screening are less effective than studies with recruitment through referral. The authors suggest some explanations that might apply to our study. Patients who do not actively seek treatment might have good reasons for not seeking treatment themselves. They might be different from those who actively seek treatment in a number of ways but these differences are as yet unknown. The patients in both stepped care and care as usual had a, on average, mild to moderate symptom levels, but the small change in symptoms over time and the high number of existing diagnosis at the end of the study suggests that we included a chronic group of patients. Further evidence for the chronicity in the study sample can be seen in that the mean age of the sample is 50 years but the mean age of onset is 28, suggesting an average 22 year chronicity. This problem could be eliminated with referral through the GP by referring patients that would benefit from a stepped care model and refer patients with chronic mental problems to specialized mental health care. Therefore stepped care can be applied for eligible patients or for the prevention of anxiety and/or anxiety, which is already been proven effective for elderly [34]. Prevention with low-intensity treatment could reduce the development of a full-blown disorder.

Patients with relatively mild symptoms might have less room for improvement compared to those with severe symptoms and they also might be less motivated for treatment and therefore show no decrease of symptoms. This also could explain why the adherence to the stepped care program was poor, in total 38 (63.3%) of 60 patients dropped out of the stepped care model at a given time. Certainly for the self-help step, in this step most patients dropped out of the program. Apart from lack of motivation, the freedom of choice for feedback may have led to no feedback requests. In previous research it has been established that self-help without guidance is not effective. It is highly recommended to give more attention to the guidance of the self-help course [65]. In our study patients could choose if they wanted to receive feedback on their assignments, but there is evidence that in interventions without a coach, compared to interventions with a coach, the drop-out rate is considerably higher [66]. To create a better adherence to the self-help course, guidance by a coach should be considered. Another suggestion for adjustment to create higher adherence to the care model is to search for other low-intensity treatments as a first step and, for example, give the patient a choice between two or more treatments. In the Phase IV field trial, described by Richards and Suckling [67], they combine low-intensity stepped care psychological treatment with a telephony-based collaborative care organizational system. This would be a more flexible approach to stepped care.

Our suggestion to improve the model would be to give the patient a choice in the first step between low-intensity treatments, for example: (guided) self-help and psycho-education in few group-sessions or they might even choose to skip the first step. This psychoeducational group therapy (with: patient's education, behavioural activation, problem solving techniques) has been an effective first step in a randomized controlled trial of treating severe depression in primary care with a stepped care program [68]. At last, the care as usual in The Netherlands is quite well developed in terms of evidence-based guidelines, mental health specialists working in primary care and it is easily accessible for patients. Therefore, this study was not a placebo control but has a good quality control group. This might have led to no differences between both groups and it might be that a stepped care model is not effective for use in Dutch primary care.

Through the earlier mentioned problem with recruiting patients for this study the power of this study was also a limitation. We intended to include 200 patients, but because of the problematic recruitment we only were able to include 120 patients. Nevertheless we do not think that an inclusion of 200 patients would have changed our results given the small effects.

## Conclusions

In summary we could not demonstrate that stepped care for depression and anxiety in general practice was more effective than care as usual. This model, with recruitment through screening in patients with mild disorders is not a good methodology. For further research on stepped care we recommend recruitment of patients via referral of the GP. Studies on care models and complex interventions are of increasing importance because they provide effective health care. More research is needed on the development of mental health care models that fit into the local care system.

## Competing interests

The authors declare that they have no competing interests.

## Authors' contributions

AvS and PC obtained funding for the study. WS coordinated the recruitment and data collection during the study. AB and PC are responsible for the overall supervision. HvM, AvS and PC provided the setting of the project. All authors provided comments, read and approved the final manuscript.

## Financial Disclosure

This study is funded by ZonMw - the Dutch Organisation for Health Research and Development (Zorg Onderzoek Nederland, ZonMw), grant-number: 10003020.
